# Modification of Spherical Polyelectrolyte Brushes by Layer-by-Layer Self-Assembly as Observed by Small Angle X-ray Scattering

**DOI:** 10.3390/polym8040145

**Published:** 2016-04-15

**Authors:** Yuchuan Tian, Li Li, Haoya Han, Weihua Wang, Yunwei Wang, Zhishuang Ye, Xuhong Guo

**Affiliations:** 1 State-Key Laboratory of Chemical Engineering, East China University of Science and Technology, Shanghai 200237, China; tianyuchuanecust@163.com (Y.T.); 020120096@mail.ecust.edu.cn (H.H.); wang88101500@163.com (W.W.); Y20140094@mail.ecust.edu.cn (Y.W.); 18817507593@163.com (Z.Y.); 2 Engineering Research Center of Xinjiang Bingtuan of Materials Chemical Engineering, Shihezi University, Xinjiang 832000, China

**Keywords:** spherical polyelectrolyte brushes, layer-by-layer deposition, small X-ray scattering

## Abstract

Multilayer modified spherical polyelectrolyte brushes were prepared through alternate deposition of positively charged poly(allylamine hydrochloride) (PAH) and negatively charged poly-l-aspartic acid (PAsp) onto negatively charged spherical poly(acrylic acid) (PAA) brushes (SPBs) on a poly(styrene) core. The charge reversal determined by the zeta potential indicated the success of layer-by-layer (LBL) deposition. The change of the structure during the construction of multilayer modified SPBs was observed by small-angle X-ray scattering (SAXS). SAXS results indicated that some PAH chains were able to penetrate into the PAA brush for the PAA-PAH double-layer modified SPBs whereas part of the PAH moved towards the outer layer when the PAsp layer was loaded to form a PAA-PAH-PAsp triple-layer system. The multilayer modified SPBs were stable upon changing the pH (5 to 9) and ionic strength (1 to 100 mM). The triple-layer modified SPBs were more tolerated to high pH (even at 11) compared to the double-layer ones. SAXS is proved to be a powerful tool for studying the inner structure of multilayer modified SPBs, which can establish guidelines for the a range of potential applications of multilayer modified SPBs.

## 1. Introduction

In recent years, polyelectrolyte multilayer nanoparticles prepared by layer-by-layer (LBL) deposition of oppositely charged polyelectrolytes onto various templates have attracted great scientific interest [1,2,3]. Because of the unique advantage of their multi-functionality, they are ideal candidates in various areas such as drug delivery [4,5,6,7,8], surface modification [9,10,11,12], biosensors [13,14], photonic devices [15] and nanoreactors [16].

Different kinds of nanoparticles (NPs) [17,18], such as liposomes [19,20], hydrogels [21], silica [22,23], and calcium salt [4], can be used as the templates to obtain the multilayer structures. Spherical polyelectrolyte brushes (SPBs) [24,25,26,27,28,29], in which polyelectrolyte chains are attached onto the surface of spherical cores covalently by one end, were also reported as one of the ideal templates of polyelectrolyte multilayer nanoparticles [16]. Compared with other conventional templates for multilayer structures, SPBs have a unique brush structure to enable a larger loading rate of the functional molecules or nanoparticles, and the brush layers are very sensitive to the environmental factors, such as pH, ionic strength or temperature, which makes it easy to control the exchange of substances inside and outside the multilayer system, providing an immense potential in the applications of controlled drug release, high-efficiency nano-reactors, and high-selectivity biosensors. However, most of the publications on SPBs are mainly focused on the applications of SPBs as nano-reactors [30,31,32], protein immobilizers [33,34,35,36], and organic-inorganic hybrids [37,38,39], and only few papers [16] concerning the multilayer modified SPBs by LBL deposition could be found, and even less attention was paid to their structural change during construction and under various environments (e.g., pH and ionic strength). It is imperative to understand and control the properties of multilayer modified SPBs in order to give better instructions on their applications. Thus, this study is aimed at the structural analysis of the multilayer modified SPBs.

Among all the methods to study the structure of NPs, small-angle X-ray scattering (SAXS) has been proven to be one of the most powerful ways [40,41,42,43,44,45]. SAXS is especially capable of analyzing the sample *in situ* within various environments in a mild condition where less destruction is caused to the system. Moreover, SAXS could give a detailed illustration of the inner structure and size distribution of the NPs, whereas using other methods such as TEM, only the overall morphology could be obtained and the details of the nanostructure are inevitably missing.

**Figure 1:**
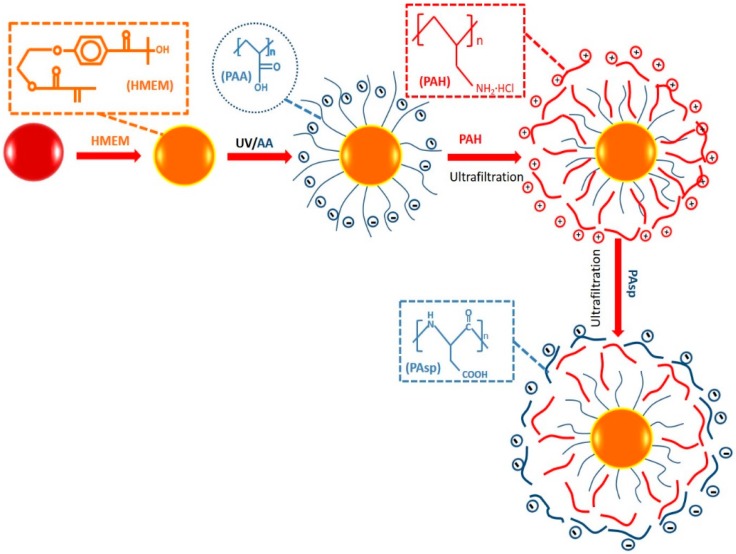
Schematic illustration of the modification of SPBs by layer-by-layer self-assembly.

Herein we present the study of the structural features of the multilayer modified SPBs during the LBL deposition and at different pH values and ionic strengths mainly using SAXS. As illustrated in Figure 1, multilayer modified SPBs were prepared by the alternate LBL deposition of positively charged PAH and negatively charged PAsp onto negatively charged PAA brushes. During the construction of multilayer modified SPBs, transmission electron microscopy (TEM) was employed to see the NPs’ morphology, zeta potential was adopted to observe the charge reversal, DLS (Dynamic Light Scattering) was used to measure the overall size change, and SAXS was employed to observe the distribution of polyelectrolytes in the multilayer modified SPBs. The five-layer excess electron density (Δρe) distribution model was used to fit the SAXS results. From the change of the excess electron density distribution of the multilayer modified SPBs, information on the inner structure and distribution of the polyelectrolytes was obtained.

## 2. Experimental Section

### 2.1. Materials

Styrene, acrylic acid (AA), K_2_S_2_O_8_ (KPS), and sodium dodecyl sulfonate (SDS) are purchased from Shanghai Reagent Company, Shanghai, China. The 2-hydroxy-4′-hydroxyethoxy-2-methyl propiophenone (HMP), and methacryloyl chloride were purchased from Tokyo Chemical Industry Co., Tokyo, Japan. Poly(allylamine hydrochloride) (PAH) (*M*_w_ = 17,000 g/mol, >99.1%), was purchased from Sigma-Aldrich (St. Louis, MO, USA). Poly-l-aspartic acid (PAsp) (*M*_w_ = 10,000 g/mol, >98%) was bought from Aike Reagent Company (Chengdu, China). Photo-initiator 2-[p-(2-hydroxy-2-methyl propiopenone)]-ethyleneglycol methacrylate (HEMEM) was synthesized in our laboratory according to our previous paper [24]. Ultrapure water was purified by reverse osmosis (Milli-Q, Millipore, MA, USA) and used in all experiments. Styrene and AA were used after reduced pressure distillation and stored in a refrigerator of 4 °C. KPS was recrystallized by water. All other materials were used without further purification.

### 2.2. Preparation of Multilayer Modified SPBs

The PS core was synthesized using a conventional emulsion polymerization. At first, 0.74 g KPS was dissolved in 150 mL water in a 500 mL three-necked round-bottomed flask, followed by adding of 0.24 g SDS dissolved in 10 mL water. Then 10 g styrene was added to the flask followed by the repeatedly degassing and subsequent addition of nitrogen at least three times. The reaction was carried out at 80 °C for 2 h under the nitrogen atmosphere with a stir rate of 300 rpm. Finally 1 g photo-initiator HMEM dissolved in 7 g acetone was slowly added to the system. To form a well-defined core-shell structure, HMEM was added under starved condition (0.05 mL/min). After another reaction for 2.5 h, the obtained PS core was purified in Milli-Q water by dialysis.

The spherical PAA brushes were synthesized using photo-emulsion polymerization. In a typical run, 100 g PS core solution were added to a 500 mL three-necked flat-bottomed quartz photo-reactor. The amount of AA in mole is equal to that of styrene in PS core. Then extra amount of deionized water was added to the reactor until the mass of the whole reaction system reached 400 g. After adding AA to the PS core latex, the system was degassed by repeatedly evacuating and adding nitrogen at least three times. Then photo-emulsion polymerization was accomplished with UV radiation at room temperature with vigorous stirring for 2.5 h. The obtained SPBs were purified by ultra-filtration until the conductivity of outer water became constant.

The obtained PAA brushes were diluted to 10 mg/mL and adjusted to pH 7 by 0.1 M NaOH solution. Then aqueous solutions of PAH and PAsp were adjusted to the same concentration (10 mg/mL) and pH (pH = 7). To form a stable PAA-PAH double-layer structure, 25 mL of PAA brushes were added to 25 mL PAH solution under the “starved condition” with vigorous stirring, after that an ultrafiltration with 2 L water was conducted to remove the excessive PAH chains in the system. Then the PAA-PAH double-layer modified SPBss were obtained. Finally, PAsp was deposited onto the PAA-PAH modified SPBs with the similar manner. After the ultrafiltration with 2 L water, the PAA-PAH-PAsp triple-layer modified SPBs were obtained.

### 2.3. Characterization

The morphology of SPBs and multilayer modified SPBs was observed by transmission electron microscopy (TEM) (JEM-2010, JEOL Ltd., Tokyo, Japan). The hydrodynamic size of SPBs and multilayer modified SPBs was determined by dynamic light scattering (DLS) (Particle Sizer NICOMP 380 ZLS, Particle Sizing Systems, CA, USA) at variable pH and ionic strength at 25 °C and at a scattering angle of 90°.

The apparent zeta potential ξ was estimated from Equation (1): [46]

(1) $$\zeta =\frac{4\pi \eta }{\varepsilon }\bar{\mu }$$

where ε and η are the permittivity and viscosity of the dispersion medium, respectively. The mean electrophoretic mobility $\bar{\mu }$ was determined by NICOMP 380 ZLS (Particle Sizing Systems, CA, USA) by Electrophoretic Light Scattering based on Phase Light Scattering (PALS) [46]. When electric field was added to the test cell, charged NPs would move towards the oppositely charged electrode.

All the SAXS data were collected at beamline BL16B1 in Shanghai Synchrotron Radiation Facility (SSRF). During the SAXS test, 0.1 mL of SPBs solution were placed at the groove of a 1-mm-thick polystyrene plastic template wrapped by polyimide films on both sides.

### 2.4. SAXS Fitting Model

The scattering intensity *I*_0_ was presented as a function of *q* to fit the SAXS scattering curve. Here *q* means scattering vector which depends on the scattering angle θ:

(2) $$q=\frac{4\pi }{\lambda }\sin\frac{\theta }{2}$$

For a single particle, the scattering intensity *I*_1_(*q*) is related with the particle volume *V*_1_ and its excess electron density Δρ_e_:

(3) $$I_{1}(q)=V_{1}{}^{2}\Delta \rho_{e}{}^{2}P(q)$$

For the monodisperse particle system, the scattering intensity *I*(*q*) can be further described as following:

(4) $$I_{1}(q)=NV_{1}{}^{2}\Delta \rho_{e}{}^{2}P(q)S(q)$$

Here *P*(*q*) is the form factor which contributes to the intraparticle part of the scattering intensity, whereas *S*(*q*) is the structure factor which denotes the interparticle part. In the case of a dilute system (≤1 wt %), the structural factor can be approximated to one.

As for a dilute polydisperse system (where *S*(*q*) = 1), *I*(*q*)) can be derived by the sum of the scattering intensity *I*_1_(*q*) of every single particle, *i.e.*,:

(5) $$I(q)=\sum_{i=1}^{N}{\left(\Delta \rho_{e}\right)}_{i}^{2}V_{i}{}^{2}P_{i}\left(q\right)$$

As for a single SPB particle, the scattering intensity can be divided into three independent parts [25]:

(6) $$I_{1}\left(q\right)=I_{\text{fluct}}\left(q\right)+I_{\text{ps}}\left(q\right)+I_{\text{cs}}\left(q\right)$$

Here the first term *I*_fluct_(*q*) stands for the fluctuation of the polyelectrolyte chains in the system (including the PAA chains attached to the PS core and the PAH or PAsp chains adsorbed in the shell of the SPBs), which mainly influences *I*(*q*) at high scattering angles.

(7) $$I_{\text{fluct}}(q)=\frac{I_{\text{fluct}}(0)}{1+\xi^{2}q^{2}}$$

where ξ is the correlation length of the spatial fluctuations of the polyelectrolyte chains. In this context both *ξ* and *I*_fluct_(0) are adjustable parameters.

The second part *I*_ps_(*q*) denotes the part of the fluctuation within the PS core, which is far less than the other two parts and thus could be neglected in most cases.

The last part *I*_cs_(*q*) is caused by the contrast of the electron density between the core-shell structure and the surrounding medium (between PS core and water or PAA chains and water), which could be described as following through Fourier transform [25]:

(8) $$I_{cs}(q)=B^{2}(q)={\left[4\pi \int_{0}^{R}\Delta \rho_{e}(r)\frac{\sin(qr)}{qr}r^{2}dr\right]}^{2}$$

*I*_cs_(*q*) is an important factor that could “project” the structure of a single SPB particle in real space which only influences *I*(*q*) at small scattering angles.

As a result, the key to fit *I*_cs_(*q*) is to find an appropriate model to signify the distribution of Δρ^e^(*r*) in the radical direction. Among these different kinds of theoretical distribution models of Δρ^e^(*r*) [25,43,44,47,48,49,50,51,52,53], firstly developed by Rosenfeldt [52], the five-layer model [25,43,44,52,53] has proven to be an effective model to simplify the Δρ^e^(*r*) without sacrificing the accuracy of the model, especially for more complicated systems when SPBs interact with other charged groups, such as proteins, polyelectrolytes, ions, *etc.*, and both the distribution of the polyelectrolyte chains of the brushes and other charged groups within would vary significantly under different conditions (concentration, pH, and ion strength, *etc.*) because of the electrostatic effect.

**Figure 2:**
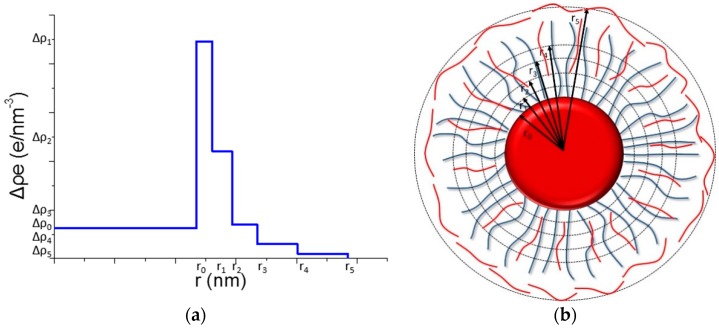
The five-layer model of the distribution of Δρ^e^ for multilayer SPB. (**a**) Schematic illustration of multilayer SPB; (**b**) Illustration of the five-layer model for multilayer modified SPBs with a core of PS, the red lines, PAH, gray lines, PAA chians.

As shown in Figure 2a, the distribution of the shell is divided into five layers (1 ≤ *i* ≤ 5). At each layer, the excess electron distribution is simplified to a particular constant Δρ_i_. Additionally, Δρ_0_ stands for the excess electron density of the homogeneous PS core (6.4 e/nm^3^). Here *r*_0_ stands for the radius of the PS core, and *r*_i_ stands for the radial distance from the center of the PS core to the edge of the “*i*^th^” layer.

For each different part, *B*_i_(*q*) can be calculated through the integral of Equation (8) [43]:

(9) $$B_{i}(q)=\left\{ \begin{array}{l} \Delta \rho_{0}{}^{e}(\frac{\sin qr_{0}-qr_{0}\cos qr_{0}}{q^{3}})\;(i=0)\\ B_{i}(q)=\sum_{j=i-1}^{i}\Delta \rho_{j}{}^{e}(\frac{\sin qr_{j}-qr_{j}\cos qr_{j}}{q^{3}})\;(1\leq i\leq 5) \end{array}\right.$$

The scattering amplitude of the core-shell structure *B*_cs_(*q*) is the sum of each different part.

(10) $$B_{\text{cs}}(q)=\sum_{i=0}^{5}B_{i}(q)$$

As for the polydisperse SPBs system, Gaussian distribution *G*(r) was used to describe the size distribution of the PS core and PAA chains, respectively.

(11) $$G(R,R_{0},\sigma )=\frac{1}{\sigma \sqrt{2\pi }}\exp(-\frac{{\left(R-R_{0}\right)}^{2}}{2\sigma^{2}}),\;\int_{0}^{\infty }G(r)d(r)=1$$

*R*_0_ is the average radius (for PS core *R*_0_ = *R*_core_, for PAA shell *R*_0_ = *R*_shell_), and *σ* is the stand deviation. When combining Equations (6)–(11), the scattering intensity *I*(*q*) of the SPB system can be obtained through Equation (12).

(12) $$I(q)=\gamma \frac{N}{V}I_{1}(q)=\gamma \frac{N}{V}(\int_{0}^{\infty }\int_{0}^{\infty }G(r_{c})G(r_{s})(\sum_{i=1}^{5}B_{i}{\left(q\right)}^{2})d(r_{c})d(r_{s}))$$

Here γ is a constant independent of *q* and *r*, and *N*/*V* is the number density.

For the SPB loaded with PAH and PAsp, because the adequate amount of water (approximately 40 times the volume of original multilayer modified SPBs solution) was used in the process of ultrafiltration to remove the unabsorbed polyelectrolyte chains, almost all the unabsorbed polyelectrolyte chains were erased from the system. Thus, the scattering intensity *I*(*q*) of the multilayer system is contributed only from the SPBs loaded with polyelectrolyte chains.

When loaded with different kinds of polyelectrolytes, the excess electron density of the multilayer modified SPBs would be significantly increased (Table 1), thus changing the scattering intensity *I*(*q*). Through the established model, the distribution of polyelectrolytes under various conditions can be “observed”.

It is worth noting that, for each sample, the scattering intensity is normalized to the absolute intensity in agreement with pure water. Additionally, by subtracting the background *I*_cell_ and *I*_water_ and the noise *I*_dark_ from the measured *I*(*q*), the scattering intensity of only the particles is obtained.

## 3. Results and Discussion

### 3.1. Structure of Multilayer SPB

#### 3.1.1. By TEM

**Figure 3:**
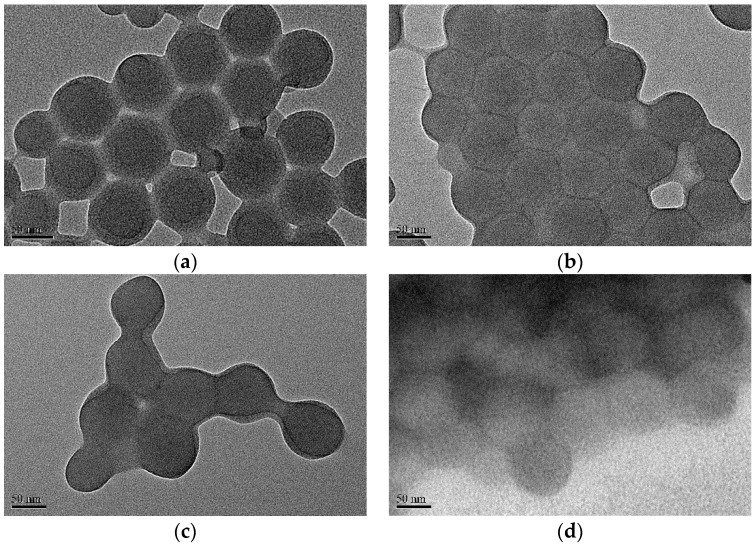
TEM images of (**a**) PS core; (**b**) SPB; (**c**) PAA-PAH double-layer modified SPB; and (**d**) PAA-PAH-PAsp triple-layer modified SPB.

In Figure 3, the morphologies of the PS core, SPB, PAA-PAH double-layer SPB, and PAA-PAH-PAsp triple-layer SPB were observed by TEM. After the alternate deposition of the polyelectrolytes, the multilayer modified SPBs remained in a spherical shape. However, the brush layers cannot be determined. It seems that the resolution of the TEM images became worse after more polyelectrolytes were deposited, especially for the PAA-PAH-PAsp triple-layer modified SPBs (Figure 3d). Obviously, TEM is not a good method for observing the structure of the polyelectrolyte brush layer because during the sample preparation the structure has been changed significantly.

#### 3.1.2. By Zeta Potential and DLS

**Figure 4:**
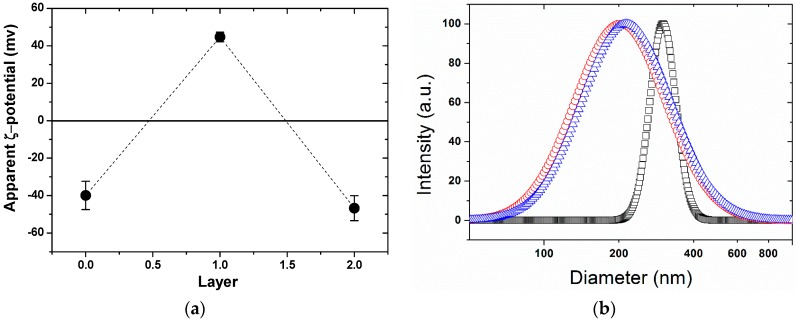
(**a**) Zeta-potential of multilayer SPBs as a function of the number of deposition layers; (**b**) DLS curves of multilayer modified SPBs. Symbol denotes: (□, black) SPBs; (○, red) PAA-PAH double-layers; (∆, blue) PAA-PAH-PAsp triple-layers. (pH = 7, C_NaCl_ = 10 mM).

To track the polyelectrolytes’ layer-by-layer deposition, the apparent zeta potentials of the multilayer modified SPBs were determined. As shown in Figure 4a, the apparent zeta potential of PAA SPB increased from −39.6 to +44.7 mv after coating with the first PAH layer, and then turned to −46.7 mv upon deposition of the second PAsp layer. The reversal of charge after each layer deposition indicated the successful preparation of multilayer modified SPBs.

As observed by DLS (Figure 4b), the hydrodynamic diameter of the SPBs decreased and their size distribution increased significantly after the deposition of PAH. This interesting size change was different from other reports on the LBL PAA/PAH multilayer using liposomes [53] or CaCO_3_ nanoparticles [54] as templates, where the diameter increased after the first layer was formed on the templates. That could be explained by the fact that the positively charged PAH chains partly penetrated into the PAA brush layer and neutralized the negative charges of the carboxyl groups of the PAA. As a result, the electrostatic repulsions between the PAA chains were weakened, which led to the shrinkage of the SPB layer. Due to the difference in the deposition amount of the PAH chains onto each SPB, the size distribution of the PAA-PAH double-layer modified SPBs became broader. As shown in Figure 4b, further coating of the negatively charged PAsp had less impact on the size distribution, though it led to a slight increase in the average diameter. It seems that the PAsp chains were mainly located on the surface of the multilayer modified SPBs.

#### 3.1.3. By SAXS

**Figure 5:**
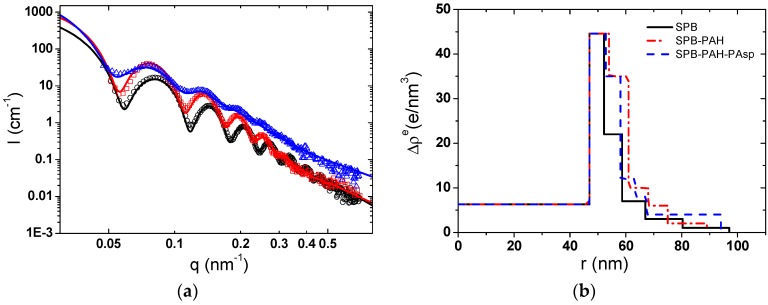
(**a**) Scattering intensity of SPBs (○, black), PAA-PAH double-layers (□, red) and PAA-PAH-PAsp triple-layers (∆, blue). The solid line is the fitting curve; (**b**) The radial profile of the excess electron density of multilayer modified SPBs as a function of the radius. (C_NaCl_ = 10 mM, pH = 7). All the SAXS curves were normalized to the same mass fraction of the SPB solution.

As observed by SAXS in Figure 5a, the scattering intensity of the SPBs increased and the first maximum of the scattering curve shifted to a smaller scattering angle during the construction of the multilayer modified SPBs, which formed the deposition of the polyelectrolyte layers. The amplitude of the oscillation decreased after the polyelectrolyte deposition which means the broadening of the size distribution and is consistent with the DLS results (Figure 4b). Moreover, at medium to high *q* values (*q* > 0.3 nm^−1^), the significant increase in scattering intensity of PAA-PAH-PAsp triple-layer modified SPBs should be due to the increased thermal fluctuation of the polymer chains located on the surface. It confirmed that the PAsp chains were mainly located on the surface of the PAA-PAH-PAsp triple-layer modified SPBs without penetrating into the shell of the PAA-PAH.

The radial profile of the excess electron density Δρ^e^ in Figure 5b reflected similar information as the SAXS scattering curves and DLS curves (Figure 4b). The increase in the electron density of inner layers for PAA SPBs after the deposition of PAH confirmed the hypothesis that some PAH chains were penetrated into the brush interior. Interestingly, the overall Δρ^e^ of the first four layers decreased when the PAsp was deposited onto PAA-PAH double-layer modified SPBs. It seems that the PAH chains penetrated in the inner part of the SPBs moved towards the outer layer due to the electrostatic attraction from the PAsp at the outmost layer.

### 3.2. Effect of pH

#### 3.2.1. PAA-PAH Double-Layer Modified SPB

**Figure 6:**
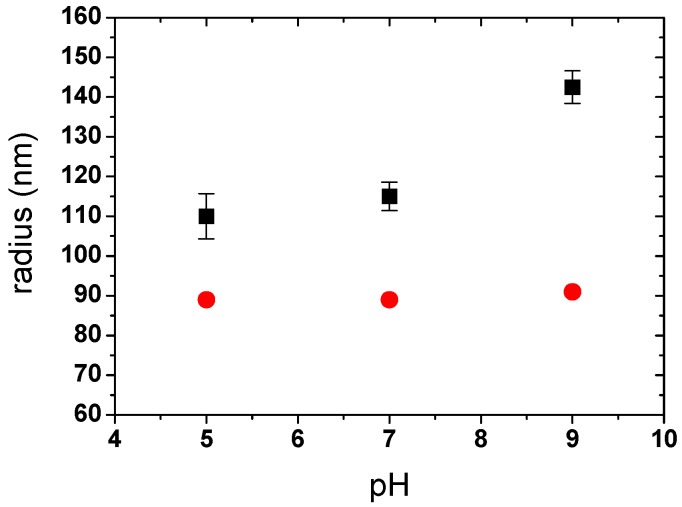
Effect of pH on the radius of PAA-PAH double-layer modified SPBs measured by DLS and SAXS (C_NaCl_ = 10 mM). Symbols denote: (■, black) DLS, (●, red) SAXS.

The radii of PAA-PAH double-layer modified SPBs obtained by both DLS and SAXS were compared in Figure 6. DLS results showed that the radius increased upon increasing pH, especially at pH 9. At a further increase in pH to 11, SPBs became unstable and aggregation was found (radius > 500 nm, polydispersity index > 0.5). Upon increasing the pH, the charge amount in the PAH decreased, which made the electrostatic attraction between PAH and PAA chains weak. Therefore, most of the PAH may move out of the SPBs, which can be determined as an increase in hydrodynamic size by DLS.

**Figure 7:**
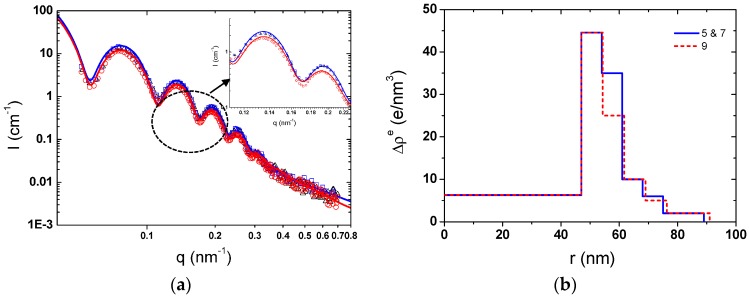
(**a**) Scattering intensity curves of PAA-PAH double-layer modified SPBs at pH 5 (∆, black), pH 7 (□, blue), and pH 9 (○, red). The solid lines are the corresponding fitting curves; the inset is the enlarged view of scattering curves (0.1 nm^−1^ < *q* < 0.23 nm^−1^); (**b**) The radial profile of excess electron density of PAA-PAH at different pH (C_NaCl_ = 10 mM).

However, the size of double-layer modified SPBs remained almost unchanged within the experimental range as observed by SAXS (Figure 6 and Figure 7a). The size measured by DLS appeared larger than that measured by SAXS because DLS measured the hydrodynamic size, which is sensitive to the longest polymer chains, while SAXS determined the radius of gyration, which depends on the mass distribution. Moreover, the concentration of the samples for the SAXS measurement was much higher than that for the DLS measurement [39].

Zooming in the SAXS curves in Figure 7a, those at pH 5 and 7 were overlapped while there was a slight increase in the scattering density at pH 9. The excess electron density of the second layer from the core decreased from 35 to 25 e/nm^−3^ (Figure 7b), which reflects the fact that most of the PAH chains were desorbed from the SPB shells due to the weakened electrostatic attraction.

#### 3.2.2. PAA-PAH-PAsp Triple-Layer Modified SPB

**Figure 8:**
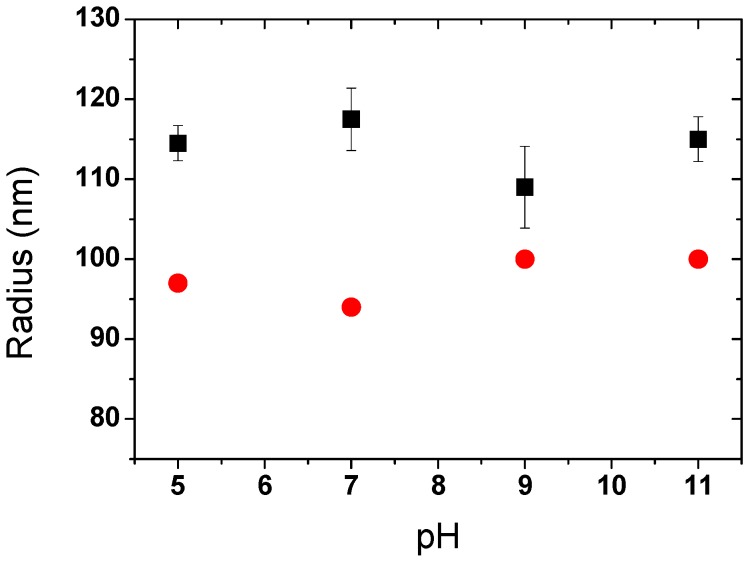
Effect of pH on the radius of PAA-PAH-PAsp triple-layer modified SPBs measured by DLS and SAXS (C_NaCl_ = 10 mM). Symbols denote: (■, black) DLS, (●, red) SAXS.

After coating the PAsp, the triple-layer modified SPBs became stable in a wider range of pHs. Upon increasing the pH from 5 to 11, the change of size for the triple-layer modified SPBs was very small, as observed by both DLS and SAXS (Figure 8). The dissociation of carboxyl groups in the PAsp layer increased the electrostatic repulsions among SPBs and thus the stability at high pH remained.

**Figure 9:**
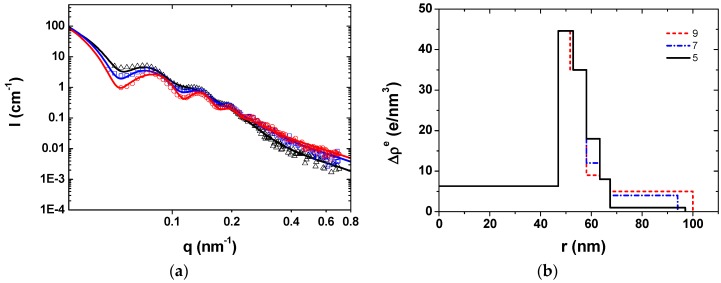
(**a**) Scattering intensity curves of PAA-PAH-PAsp triple-layer modified SPBs at pH 5 (∆, black), 7 (□, blue) and 9 (○, red). Solid lines are the fitting curves; (**b**) The radial profile of excess electron density of PAA-PAH-PAsp triple-layer modified SPBs at different pH (C_NaCl_ = 10 mM).

In Figure 9a, the SAXS curves gave more information on the change of the size distribution. Upon increasing the pH from 5 to 9, the location of the first maximum kept stable, which means the size did not change, while the increase in the oscillation amplitude reflects that the size distribution became smaller.

When the pH increased from 5 to 9, the excess electron density Δρ^e^ of the inner layer decreased while that in the outermost layer increased (Figure 9b). This means that the PAH chains were probably redistributed and moved towards the outer layer.

### 3.3. Effect of Ionic Strength

**Figure 10:**
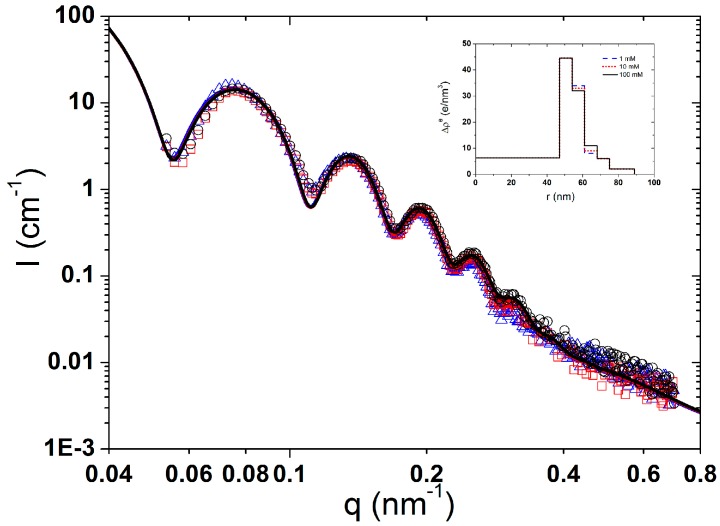
Scattering intensity curves of PAA-PAH double-layer modified SPBs at different ionic strengths. Symbols denote: (○, black) 100 mM, (□, red) 10 mM, and (∆, blue) 1 mM. Solid lines are the SAXS fitting data. The inset is the radial profile of excess electron density.

The ionic strength was controlled by the addition of aqueous NaCl concentration. In Figure 10, the SAXS curves and the radial excess electron density profiles of PAA-PAH double-layer modified SPBs at ionic strengths of 1, 10 and 100 mM were almost overlapped. It means that the ionic strength has less impact on the structure of PAA-PAH double-layer modified SPBs.

**Figure 11:**
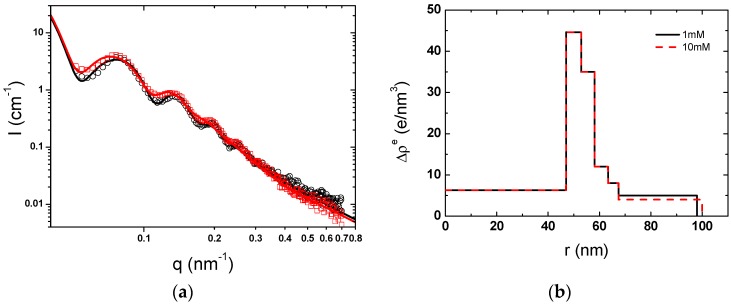
(**a**) Scattering intensity curves of PAA-PAH-PAsp triple-layer modified SPBs at NaCl concentrations of 1 mM (○, black), and 10 mM (□, red). Solid lines represent the fitting curves; (**b**) Excess electron density of PAA-PAH-PAsp at different salt concentrations.

As shown in Figure 11a, the scattering intensity of PAA-PAH-PAsp triple-layer modified SPBs was also almost unchanged, but the size distribution slightly turned to broad upon increasing the salt concentration from 1 to 10 mM.

The excess electron distribution of triple-layer modified SPBs in Figure 11b revealed that the PAH chains penetrated in the brush layer turned to move out upon increasing the ionic strength.

## 4. Conclusions

Multilayer modified PAA brushes were prepared by the alternative deposition of PAH and PAsp onto negatively charged spherical PAA brushes. The success of LBL deposition was confirmed by the charge reversal of the zeta potential and the size increase observed by DLS and SAXS. The five-layer excess electron density distribution model was used to fit the SAXS results. The SAXS results indicated that some PAH chains were able to penetrate the PAA brush during the construction of the PAA-PAH double-layer modified SPBs, while these PAHs moved towards the outer layer when the PAsp molecules were loaded to form PAA-PAH-PAsp triple-layer modified SPBs. Upon increasing the pH from 5 to 9, the size of multilayer modified SPBs was hardly changed, but the size distribution of triple-layer SPBs became narrower. The triple-layer modified SPBs kept stable even at pH 11. The average size and size distribution of both double-layer and triple-layer modified SPBs were almost unchanged upon changing the ionic strength from 1 to 100 mM. The movement of the penetrated PAH chains towards the outer layer of the triple-layer modified SPBs by increasing pH or ionic strength was observed by SAXS.

## Figures and Tables

**Table 1:** Electron density of related substances.

Substance	ρ (g/cm^3^)	ρ_i_ (e/nm^3^) ^β^	Δρ (ρ_i_-ρ_H2O_, e/nm^3^)
H_2_O	0.997 *	333.3	0
Poly(styrene)	1.05 *	339.7	6.4
Poly(arylic acid)	1.19 ^α^	377.9	44.6
Poly(allylamine hydrochloride)	1.10 ^α^	376.8	43.5
poly-l-aspartic acid	1.20 ^α^	376.9	43.6

* Mass density from Reference [45]; ^α^ Mass density measured by density meter; ^β^ Electron density from calculation.
